# The BDNF Val^66^Met Polymorphism Has Opposite Effects on Memory Circuits of Multiple Sclerosis Patients and Controls

**DOI:** 10.1371/journal.pone.0061063

**Published:** 2013-04-11

**Authors:** Francesco Fera, Luca Passamonti, Antonio Cerasa, Maria Cecilia Gioia, Maria Liguori, Ida Manna, Paola Valentino, Aldo Quattrone

**Affiliations:** 1 Università degli Studi “Magna Graecia”, Dipartimento di Scienze Mediche e Chirurgiche, Catanzaro, Italia; 2 Consiglio Nazionale delle Ricerche, Unità di Ricerca Neuroimmagini, Catanzaro, Italia; 3 Consiglio Nazionale delle Ricerche, Istituto di Scienze Neurologiche, Mangone (CS), Italia; University of Cambridge, United Kingdom

## Abstract

Episodic memory deficits are frequent symptoms in Multiple Sclerosis and have been associated with dysfunctions of the hippocampus, a key region for learning. However, it is unclear whether genetic factors that influence neural plasticity modulate episodic memory in MS. We thus studied how the Brain Derived Neurotrophic Factor Val^66^Met genotype, a common polymorphism influencing the hippocampal function in healthy controls, impacted on brain networks underlying episodic memory in patients with Multiple Sclerosis. Functional magnetic resonance imaging was used to assess how the Brain Derived Neurotrophic Factor Val^66^Met polymorphism modulated brain regional activity and functional connectivity in 26 cognitively unimpaired Multiple Sclerosis patients and 25 age- and education-matched healthy controls while performing an episodic memory task that included encoding and retrieving visual scenes. We found a highly significant group by genotype interaction in the left posterior hippocampus, bilateral parahippocampus, and left posterior cingulate cortex. In particular, Multiple Sclerosis patients homozygous for the Val^66^ allele, relative to Met^66^ carriers, showed greater brain responses during both encoding and retrieval while the *opposite* was true for healthy controls. Furthermore, a robust group by genotype by task interaction was detected for the functional connectivity between the left posterior hippocampus and the ipsilateral posterior cingulate cortex. Here, greater hippocampus-posterior cingulate cortex connectivity was observed in Multiple Sclerosis Met^66^ carriers relative to Val^66^ homozygous during retrieval (*but not* encoding) while, again, the *reverse* was true for healthy controls. The Val^66^Met polymorphism has opposite effects on hippocampal circuitry underlying episodic memory in Multiple Sclerosis patients and healthy controls. Enhancing the knowledge of how genetic factors influence cognitive functions may improve the clinical management of memory deficits in patients with Multiple Sclerosis.

## Introduction

Episodic memory deficits represent one of the most frequent cognitive symptoms in Relapsing-Remitting Multiple Sclerosis (RR-MS) and have a devastating impact on patients' ability to maintain independent living and working skills [1]–[2].

A number of previous studies have attempted to identify factors that predict the decline on episodic memory tasks in RR-MS patients [3]–[5]. Diffuse demyelination of the white-matter and the progressive degeneration across the neocortex are important causes of memory deficits in RR-MS because they tend to disrupt the communications between large scale brain networks [3]. At the same time, however, MS pathological changes stimulate neuronal plasticity that represents a fundamental adapting mechanism for maintaining a relatively normal cognitive function in spite of brain damage [6]–[12]. For example, two recent functional magnetic resonance imaging (fMRI) studies identified compensatory hyperactivations within the hippocampal system as a mechanism for preserving episodic memory in MS [13]–[14].

Previous research has also demonstrated that common variations in the DNA sequence regulate memory function at the brain and behavioural level [15]–[19]; hence, genetic factors are likely to influence the expression of cognitive symptoms in RR-MS.

The Brain Derived Neurotrophic Factor (BDNF) is critically involved in neuronal survival and is considered as the most important neuromodulator of episodic memory [20]–[24]. The non-conservative amino-acid substitution at codon 66 (Valine to Methionine, Val^66^Met) of the BDNF gene is a common polymorphism known to interfere with intracellular trafficking of the peptide and synaptic plasticity in the hippocampus [20], [24]. The relevance of BDNF in MS has also been highlighted by studies reporting that, in active MS lesions, immune cells release several neurotrophic factors including BDNF [25]–[28]. This suggests that even in the context of inflammation BDNF promotes plastic capacities of the brain [25]–[26], [28].

Enhancing our knowledge of the contribution of the BDNF polymorphism on brain activity of RR-MS patients would help defining the neurophysiological correlates underlying episodic memory in RR-MS. To this aim, neuroimaging has a unique potential for characterizing how the BDNF Val^66^Met polymorphism influences memory systems in RR-MS patients [29]–[31]. Previous fMRI studies in healthy controls have shown that the BDNF Val^66^Met polymorphism significantly alters the hippocampal activity during episodic memory tasks [22], [32]. An effect of the BDNF polymorphism has also been reported on the hippocampal volume of healthy controls and patients with psychiatric disorders [33]–[37], although a recent genome wide meta-analysis has not confirmed this finding [38].

To date, the attempts to investigate the effect of the BDNF Val^66^Met polymorphism on the brain of MS patients have produced mixed results. A first study found that RR-MS patients with at least one copy of the Met^66^ allele display reduced total grey-matter volume relative to RR-MS Val^66^ homozygous and healthy controls (HC) carrying the Met^66^ allele or homozygous for the Val^66^ allele [27]. In contrast, later studies found the opposite result (i.e., the RR-MS Met^66^ carriers had greater grey-matter volumes relative to RR-MS Val^66^ homozygous) and suggested that the Met allele may be a protecting factor for grey-matter preservation in MS [29]–[31]. Another fMRI study reported a differential effect of the BDNF polymorphism on the prefrontal-parietal activations and hippocampal disengagement in RR-MS patients and HC during a working-memory task [39]. However, this experiment did not specifically assess the contribution of the BDNF genotype on the hippocampal activity related to episodic memory.

The present study aimed at investigating the effect of the BDNF Val^66^Met polymorphism on neural systems underlying episodic memory in RR-MS patients and HC. Overall, we hypothesized a significant modulation of the BDNF Val^66^Met polymorphism on the brain responses associated to an episodic memory task that involves encoding and retrieving visual stimuli [22]. In particular, differences were predicted in the hippocampus and posterior cingulate cortex (PCC), two fundamental regions for episodic memory [13]–[14], [22], [32], [40]–[43]. At the same time, functional connectivity between the same brain regions was thought to be significantly influenced by the BDNF Val^66^Met polymorphism. BDNF is a key modulator of synaptic plasticity while MS pathology mainly affects the white-matter bundles linking different brain regions; hence, we expected to found a ‘disconnection syndrome’ as a function of the BDNF genotype and disease status.

## Participants and Methods

### MS patients and healthy controls

From a sample of 50 RR-MS patients [44] enrolled in a larger research project [27], [45], 26 patients (17 females, 9 males) were selected and classified in two groups, based on their BDNF Val^66^Met polymorphism. All patients were able to complete the fMRI task and met the following criteria: (1) no evidence of major depression or other psychiatric disorders according to the Structured Clinical Interview of the DSM-IV [46]; (2) no past or current history of traumatic brain injury or other coexisting medical disorders; (3) no clinical relapses for at least 3 months prior to study entry; (4) no assumption of antidepressant, anxiolytic, antipsychotic or antiepileptic drugs; (5) abstention from smoking and drinking caffeinated beverages for at least 6 hours before the scan; (6) no assumption of steroids and/or disease-modifying therapy in the 3 months before the study; (7) no evidence of cognitive impairment as evaluated by a detailed neuropsychological assessment (see **section 3.2**); (8) expanded disability status scale (EDSS) ranging from 1 to 2.5 [47]; (9) right handedness according to the Edinburgh handedness inventory [48]; (10) completely normal functioning of the right upper limb and optimal visual acuity. A neurologist (P.V.), blind to any other result and with more than 20 years of clinical experience, collected the clinical data from all RR-MS patients.

Twenty-five right-handed healthy controls (HC)(16 females, 9 males) with no past history of neurological or psychiatric disorders and with normal MRI of the brain (as assessed by a structural MRI scanning) were matched for age, education and Val^66^Met BDNF genotype to RR-MS patients.

Hence, there were 4 groups: (1) homozygous Val/Val (Val^66^) RR-MS patients (n = 12); (2) heterozygous Val/Met (Met^66^) RR-MS patients (n = 14); (3) homozygous Val/Val (Val^66^) HC (n = 11); (4) heterozygous Val/Met (Met^66^) HC (n = 14). To reduce the possibility of spurious effects caused by ethnical stratification, our sample only included individuals of European ancestry, both born and educated in Italy.

All participants gave written informed consent to participate in the present study, approved by the Ethical Committee of the University ‘Magna Graecia’ of Catanzaro according to the declaration of Helsinki.

### Neuropsychological assessment

RR-MS patients and HC completed a series of neuropsychological tests that were administered by a clinical neuropsychologist (M.C.G.), blind to any other result. The following cognitive functions were evaluated: (1) Intelligence Quotient (IQ)(Wechsler Adult Intelligence Scale revised, WAIS-R; IQ, total score) [49]; (2) information processing speed (WAIS-R Digit Symbol) [49]; (3) executive functions (Modified Card Sorting Test, Correct Answer, MCST-CA, and Perseverative Errors, MCST-PE) [50]; (4) verbal memory (Rey Auditory-Verbal Learning Test, RAVLT) [51]; (5) visuo-spatial skills (Benton Judgment Line Orientation Test, JLO) [52]; (6) visuo-spatial memory (Rey-Osterrieth Complex Figure Test, ROCFT) [53]; (7) working-memory (WAIS-R Digit Span Forward and Backward) [49]; (8) verbal fluency (Controlled Oral Word Association Test, COWAT) [54].

As previously reported [39], [55]–[59], RR-MS patients who failed 0, 1 or 2 tests were classified as cognitively unimpaired and included in the study. A test was considered as failed if its score was lower than the corresponding cut-off reported for the Italian normative data.

Although none of the participants met the criteria for major depression and anxiety disorders, we further investigated the presence of depressive and anxious symptoms using the Chicago Multiscale Depression Inventory (CMDI) and the Hamilton Anxiety Scale, respectively [60]–[61].

### Genotyping

DNA was extracted from peripheral blood cells using standard procedures. The G→A substitution causing the Val^66^→Met^66^ amino-acid substitution was assayed by polymerase chain reaction (PCR) amplification using the forward 5′-ACTCTGGAGAGCGTGAATGG-3′ and reverse 5′-ACTACTGAGCATCACCCTGGA-3′ primers. The amplification conditions were initiated at 95°C for 5 min., followed by 35 cycles consisting of denaturation at 95°C for 1 min., annealing at 60°C for 30 sec. and extension at 72°C for 1 min., with a final extension step of 10 min. at 72°C. Next, digestion with the Eco72I restriction enzyme was performed on PCR products and followed by a 3.0% agarose gel electrophoresis. The A allele (Met^66^) was identified as an uncut band of 171 base pairs (bp) while the G allele (Val^66^) was constituted by two cut bands, 99 and 72 bp long. A molecular biologist (I.M.) provided the BDNF genotype determinations and the doubtful results were confirmed by direct sequencing of PCR products (both directions) on an Applied Biosystems 3100 Genetic Analyzer using the Big Dye terminator cycle-sequencing reaction kit (Foster City, CA, USA).

### Magnetic Resonance imaging (MRI) acquisition

MRI scans were performed on a 1.5 T Unit using a standard quadrature head coil (Signa NV/i, General Electric, Milwaukee, WI, USA). Participants were positioned to lie comfortably in the scanner with a forehead restraining strip and various foam pads that minimized head movements during scanning.

Proton density and T2-weighted images were acquired using a conventional dual spin-echo sequence (TR = 3500 ms, TE = 20/85 ms), while T1-weighted images were obtained with a spin-echo sequence (TR 550 ms, TE 13 ms). All 2D images were acquired as axial oblique contiguous 4-mm slices (frequency/phase encoding matrix 256×256, 24 cm field of view) oriented along the anterior–posterior commisure line. A 3D T1-weighted high resolution spoiled gradient echo sequence was also acquired (TR 15 ms, TE 6.7 ms, TI 500 ms; flip angle 15°, frequency/phase encoding matrix 256×256) yielding an image volume of 70 slices, 3 mm-thick. This last sequence provided an optimal image contrast between grey-matter (GM), white-matter (WM), and cerebrospinal fluid.

For fMRI, an echo-planar image (EPI)(TR 2500 ms, TE 45 ms; flip angle 90°) was employed, with 30 axial slices of 4-mm thickness and 1-mm inter-slice gap. Slices were prescribed inferior to superior onto a mid-sagittal section.

### fMRI task

All participants executed a modified version of an episodic memory paradigm known to reliably engage the hippocampal memory network [22]. The task included 3 types of trials: (1) Encoding: subjects were presented, for 2.5 sec., with a set of coloured photographs displaying different scenes and were required to identify, via pressing a two-choice button box response, which image was “indoor” or “outdoor”; (2) Retrieval: as before, subjects saw, for 2.5 sec., “indoor” or “outdoor” pictures but now they were asked to indicate which scene was “old” (i.e., seen during encoding) or “new” (i.e., not seen during encoding); (3) Baseline: participants were shown, for 2.5 sec., a series of numbers and were required to perform an odd/even discrimination task. We employed this active baseline because there is evidence that it guarantees a more appropriate comparison task in paradigms assessing episodic memory than passive rest (e.g., fixation of a cross) [62].

A 2.5-sec. instruction period (i.e., “outdoor”/“indoor”?, “new”/“old”?, “odd”/“even”?) preceded the presentation of 11 consecutive trials of each type that were grouped in blocks lasting 30 sec. There were 3 encoding (E) blocks followed by 3 retrieval (R) blocks both alternated to 7 baseline (B) blocks (i.e., B-E-B-E-B-E-B-R-B-R-B-R-B; total task duration: 6 min., 30 sec.).

Stimuli were projected onto a back projection screen throughout a LCD video-projector while reaction times (RT) and responses at each trial were recorded via an MRI compatible fiber optic button box response controlled by LabVIEW (National Instruments, Austin, TX, USA, http://www.ni.com/labview/i/).

Mean RT and accuracy for each block type were entered in analyses of variance (ANOVA) models assessing: (1) the main effect of group; (2) the main effect of genotype; (3) the group by genotype interaction.

### Structural MRI analysis

An author (M.L.), unaware of any other result, processed the structural data. The T2-weighted total lesion load (TLL) quantification was performed on the proton density T2-weighted and T1-weighted images using a fully automated threshold technique (EMS, Medical imaging computing, Leuven, Belgium) [63]. The 3D T1-weighted images were analysed using SIENAX (Structural Image Evaluation using Normalization of Atrophy), a validated and fully automated method [64]–[65]. This technique, after deskulling, segmentation and normalization of the whole-brain volumes, calculates an estimate of global grey-matter (GM) and white-matter (WM) volumes. Furthermore, voxel-wise GM volume was assessed using Voxel Based Morphometry (VBM) methods as described in our previous research [66].

Two-sample t-tests were used to compare significant differences in TTL between RR-MS Val^66^ homozygous and RR-MS Met^66^ carriers while ANOVA models for global and voxel-wise GM and WM volumes were used to assess: (1) the main effect of group; (2) the main effect of genotype; (3) the group by genotype interaction.

### fMRI analyses

#### Preprocessing

fMRI raw data were preprocessed using SPM8 (www.fil.ion.ucl.ac.uk/spm/). The mean EPI was computed for each participant and visually inspected to ensure that none showed excessive signal dropout in the medial temporal lobes. All EPIs were next realigned to the 1^st^ scan by rigid body transformations to correct for head movements. Next, EPIs were normalized to the standard template in the MNI space (Montreal Neurological Institute (MNI)—International Consortium for Brain Mapping) using linear and non-linear transformations, and smoothed with a Gaussian kernel of full width half maximum 8-mm.

#### fMRI analyses of regional effects

These analyses aimed at: (1) identifying the effect of the BDNF Val^66^Met polymorphism on regional brain responses during episodic memory in RR-MS patients and HC; (2) obtaining reference coordinates to define the hippocampus as ‘source’ region for connectivity analyses (see **section 3.7.3.**). To these ends, a random effects model was implemented using a two-stage process (1^st^, and 2^nd^ level). This random-effects analysis allows inferences about the general population from which participants are drawn [67]. For each subject, we used a General Linear Model (GLM) to assess regionally specific effects of task on Blood Oxygenated Level Dependent (BOLD) contrast [67]. The model included 3 experimental factors (encoding, retrieval, baseline trials), and 6 realignment parameters as covariates of no interest to account for residual motion-related variance. Low-frequency signal drift was removed using a high-pass filter (cut-off = 128 sec.) and an autoregressive modeling [AR(1)] of temporal autocorrelations was applied. At the 1^st^ level, subject-specific contrast images were generated for each condition (encoding and retrieval) versus baseline. Task-specific contrast images were then entered into 2^nd^ level GLM-ANOVAs to investigate: (1) the main effect of group; (2) the main effect of genotype; (3) the main effect of task; (4) the group by genotype interaction; (5) the group by task interaction; (6) the genotype by task interaction; (7) the group by genotype by task interaction.

SPM maps were thresholded at P<0.05, Family Wise Error (FWE), whole-brain correction. Furthermore, given our strong *a priori* hypothesis on the hippocampus and posterior cingulate cortex (PCC) as key areas for episodic memory [14], [40]–[43], an ROI-based approach was employed for these regions that were defined using the “aal_02” atlas for automatic anatomical labeling (http://marsbar.sourceforge.net/) [68]. The statistical threshold for ROI analyses was set at P<0.05, FWE, small volume correction (svc), as previously recommended [69]–[70].

#### fMRI connectivity analyses: psycho-physiological-interactions (PPI)

We assessed the connectivity between a ‘source’ region (i.e., the hippocampus) and the rest of the brain that was modulated by the psychological context of encoding and retrieving visual scenes relative to baseline trials. This constitutes a Psychophysiological interaction (PPI) [69]. In other words, we sought to detect ‘target’ regions showing differential connectivity, according to the context, with the hippocampus. More importantly, we wanted to identify ‘target’ regions for which the change in connectivity with the hippocampus was modulated by the group, genotype, task and any interactive effect between these variables. Selection of the hippocampus as the “source” region was due to 3 reasons: (1) the hippocampus plays a fundamental role in episodic memory, as consistently demonstrated by previous research [14], [40]–[41]; (2) two studies in HC have shown that the BDNF Val^66^Met polymorphism significantly affects the hippocampal response during both encoding and retrieval [22], [32]; (3) our fMRI analyses of regional effects revealed a significant group by genotype interaction in the left posterior hippocampus (see **section 4.4.1**).

For each participant, a 5-mm sphere was created around the hippocampal coordinates derived from the group by genotype interaction analysis (Table S4 for MNI coordinates). The time-series of the BOLD response for each participant was computed using the 1^st^ eigenvariate from all voxels’ time-series within the sphere. Next, the BOLD time-series for each subject was deconvolved to estimate a ‘neuronal’ time-series for the ‘source’, using the PPIs deconvolution parameter defaults in SPM [71]. The PPI regressor-term was calculated as the element-by-element product of the hippocampal ‘neuronal’ time series and a vector coding for the main effect of task (1 for encoding and retrieval trials, separately, −1 for baseline trials). This product was re-convolved by the canonical hemodynamic response function (HRF). The statistical model also included the main effect of the task convolved by the HRF, the ‘source’ ‘neuronal’ time series, and the 6 movement parameters as effects of no interest. Subject-specific PPI models were run, and contrast images generated such as the identified ‘target’ regions were those showing a change in connectivity with the left posterior hippocampus as a function of the psychological context (encoding versus baseline, and retrieval versus baseline, separately).

The 1^st^ level PPI contrast images were next entered into 2^nd^ GLM-ANOVAs investigating the connectivity between the hippocampus and any brain region for: (1) the main effect of group; (2) the main effect of genotype; (3) the main effect of task; (4) the group by genotype interaction; (5) the group by task interaction; (6) the genotype by task interaction; (7) the group by genotype by task interaction.

Connectivity maps were thresholded at P<0.05, Family Wise Error (FWE), whole-brain correction or svc for ROIs [69]–[70].

#### fMRI analyses with behavioral covariates as effect of no interest in the GLM

To investigate the influence of those measures showing a significant main effect of group (i.e., RT during retrieval, digit symbol scores and visuo-spatial memory index) on brain responses in single regions and functional connectivity patterns, we repeated the analyses described in sections **3.7.2** and **3.7.3** including the behavioral covariates as effect of no interest in the GLM. The same statistical thresholding procedure described before was also applied to this set of analyses.

## Results

### Clinical and Neuropsychological results

**Table 1** reports demographic and clinical characteristics of RR-MS patients and HC. RR-MS and HC groups (i.e., RR-MS Val^66^ homozygous, RR-MS Met^66^ carriers, HC Val^66^ homozygous, HC Met^66^ carriers) were well-matched for age and educational level. Furthermore, the two groups of RR-MS patients did not differ in clinical variables (i.e., disease duration, EDSS, and Fatigue Severity Scale)(**Table 1** **)**.

**Table 1:** Demographic, clinical, brain structural, and fMRI behavioural data of relapsing-remitting multiple sclerosis (RR-MS) patients and healthy controls (HC).

	Measure	RR-MS Val^66^ (n = 12)		RR-MS Met^66^ (n = 14)		HC Val^66^ (n = 11)		HC Met^66^ (n = 14)		Main Effect of Group	Main Effect of Genotype	Groupby Genotype Interaction
Demographic	(Males = M; Females = F)	4 M; 8 F		5 M; 9 F		4 M; 7 F		5 M; 9 F				
		Mean	SD	Mean	SD	Mean	SD	Mean	SD	F; P values	F; P values	F; P values
	Age (years)	32.3	6.4	29.5	8.9	31.4	6.1	32.7	6.0	F = 0.0; P = 0.8	F = 0.4; P = 0.5	F = 0.5; P = 0.5
	Education (years)	11.5	3.48	10.5	2.6	13.3	2.4	11.7	3.7	F = 1.8; P = 0.2	F = 2.8; P = 0.1	F = 0.5; P = 0.5
**Clinical**	Disease Duration (months)	43.4	27.0	52.6	36.7	—		—		—	F = 1.1 ; P = 0.5	—
	EDSS	1.6	0.5	1.4	0.5	—		—		—	F = 0.1; P = 0.3	—
	Fatigue Severity Scale	29.0	13.0	22.2	12.4	—		—		—	F = 0.0; P = 0.2	—
**Brain Structural**	Total Lesion Load (cm^3^)	3.7	1.5	3.8	1.0	—		—		—	F = 0.4; P = 0.9	—
	Grey Matter Volume (ml)	837.5	35.2	820.8	59.4	847.4	33.6	840.0	26.5	F = 1.9; P = 0.2	F = 0.7; P = 0.4	F = 1.2; P = 0.3
	White Matter Volume (ml)	674.0	22.6	675.6	40.5	697.4	43.9	681.3	31.0	F = 3.0; P = 0.1	F = 0.3; P = 0.6	F = 0.6; P = 0.4

fMRI Task	Measure	Mean	SD	Mean	SD	Mean	SD	Mean	SD	F; P values	F; P values	F; P values
Encoding	Reaction Time (msec)	1146.6	163.0	1125.6	210.1	1123.6	214.6	1118.9	247.5	F = 0.2; P = 0.6	F = 0.0; P = 0.9	F = 0.2; P = 0.5
	Accuracy (%)	91.6	3.9	89.8	8.0	95.0	2.8	89.8	9.9	F = 1.9; P = 0.2	F = 1.8; P = 0.2	F = 0.6; P = 0.4
Retrieval	Reaction Time (msec)	1248.9	162.1	1187.5	199.5	1076.8	127.6	1088.0	124.9	F = 4.1; P = 0.05	F = 0.0; P = 0.8	F = 0.4; P = 0.5
	Accuracy (%)	87.4	6.2	87.7	8.3	91.7	6.4	88.7	9.2	F = 1.0; P = 0.3	F = 0.1; P = 0.7	F = 1.6; P = 0.2
Baseline	Reaction Time (msec)	860.4	76.0	861.7	150.9	847.6	144.1	762.1	116.8	F = 1.9; P = 0.1	F = 1.0; P = 0.3	F = 2.1; P = 0.2
	Accuracy (%)	96.9	3.0	96.4	2.5	98.3	1.2	96.5	4.5	F = 0.0; P = 0.9	F = 1.3; P = 0.3	F = 1.6; P = 0.2

**Legend.** Standard Deviation (SD). EDSS (Expanded Disability Status Scale).

There was no main effect of group, no main effect of genotype, or group by genotype interaction for a series of cognitive variables (i.e., IQ, executive functions, verbal memory, visuo-spatial skills, working-memory, and verbal fluency)(**Table 2**). A main effect of group, but not a genotype or a group by genotype effect, was found for the WAIS-R Digit Symbol and the ROFCT, in which HC, overall, responded more accurately than RR-MS patients (**Table 2**). Nonetheless, the lower scores of RR-MS patients versus HC on these neuropsychological tests were not sufficient to reach our threshold for cognitive impairment (i.e., failing more than 2 tests). Furthermore, only 1 patient within the Val^66^ MS group failed 2 tests (i.e., the 8.3%) while 4 patients (33.3%) failed 1 tests. Similarly, within the Met^66^ MS group, only 1 patient failed 2 tests (i.e., the 7.1%) while 4 patients (the 28.5%) failed 1 test. Overall, this means that the majority of MS patients did not fail any test, thus confirming that our group included subjects with only minimal cognitive impairment.

**Table 2:** Neuropsychological data of relapsing-remitting multiple sclerosis (RR-MS) patients and healthy controls (HC).

		RR-MS Val^66^		RR-MS Met^66^		HC Val^66^		HC Met^66^		MainEffect of Group	MainEffect of Genotype	Groupby Genotype Interaction
Function	Neuropsychological Test	Mean	SD	Mean	SD	Mean	SD	Mean	SD	F; P values	F; P values	F; P values
Global Cognition	WAIS-R IQ total score	100.4	18.5	103.5	17.9	116.1	9.4	107.2	23.2	F = 2.8; P = 0.1	F = 0.9; P = 0.3	F = 2.5; P = 0.1
Information Processing	WAIS-R Digit Symbol	7.0	2.6	8.6	2.7	11.7	2.7	10.9	3.5	F = 20.1; P = 0.001	F = 0.0; P = 0.9	F = 2.2; P = 0.1
Executive Functions	MCST-CA	5.7	0.5	5.8	0.01	5.8	0.01	5.7	0.5	F = 0.0; P = 0.9	F = 0.0; P = 0.9	F = 2.0; P = 0.1
	MCST-PE	2.0	1.9	1.5	2.5	2.9	2.0	2.6	3.1	F = 1.9; P = 0.2	F = 0.5; P = 0.5	F = 0.0; P = 0.9
Verbal Memory	RAVLT	39.9	10.3	38.2	8.3	39.5	4.2	42.7	7.9	F = 0.5; P = 0.5	F = 0.01; P = 0.9	F = 0.6; P = 0.4
Visuo-spatial skills	Benton JLO	25.2	3.1	24.7	4.0	25.4	3.1	24.9	4.0	F = 0.1; P = 0.7	F = 0.8; P = 0.4	F = 0.1; P = 0.7
Visuo-spatial Memory	ROCFT	14.1	5.5	16.9	5.7	19.7	6.6	20.1	3.3	F = 9.6; P = 0.01	F = 0.0; P = 0.8	F = 0.4; P = 0.5
Working-Memory	Digit Span Forward	5.7	1.7	6.0	1.6	7.1	1.8	6.6	2.2	F = 1.8; P = 0.2	F = 0.9; P = 0.3	F = 0.5; P = 0.5
	Digit Span Backward	4.7	1.6	5.0	1.3	5.5	2.3	6.4	3.4	F = 1.5; P = 0.2	F = 0.1; P = 0.7	F = 0.0; P = 0.8
Verbal Fluency	COWAT	29.6	7.5	28.9	9.0	29.8	12.9	35.6	8.6	F = 0.3; P = 0.6	F = 0.0; P = 0.8	F = 1.4; P = 0.2
Anxiety	Hamilton Anxiety Scale	20.5	5.0	19.9	7.2	15.5	4.6	17.2	5.5	F = 4.2; P = 0.06	F = 0.0; P = 0.8	F = 1.8; P = 0.2
Depression	CMDI	74.0	25.3	72.4	20.9	66.5	16.0	73.7	22.1	F = 0.0; P = 0.9	F = 0.2; P = 0.6	F = 1.5; P = 0.2

**Legend.** Standard Deviation (SD); WAIS-R: Wechsler Adult Intelligence Scale-Revised; IQ: Intelligence Quotient; Modified Card Sorting Test (MCST), CA: Correct Answers; PE: Perseverative Errors; Rey Auditory Verbal Learning Test (RAVLT); JLO: Judgment of Lines Orientation; ROCFT: Rey-Osterrieth Complex Figure Test; COWAT: Controlled Oral Word Association Test; CMDI: Chicago Multiscale Depression Inventory.

Finally, no main effect of group, no main effect of genotype, or group by genotype interaction was found for anxiety and depression (**Table 2**).

### fMRI behavioral performances

No main effect of group, no main effect of genotype, or group by genotype interaction were detected for reaction times (RT) and accuracy during encoding, retrieval, and baseline trials with the exception of a borderline main effect of group for RT during retrieval (F = 4.1; P = 0.055)(**Table 1**).

### Structural MRI results

The mean total lesion load (TLL) was not significantly different between the two groups of RR-MS patients (**Table 1**). There were no main effect of group, no main effect of genotype, or a group by genotype interaction for both global GM and WM estimates (**Table 1**). Furthermore, post-hoc two-sample t-tests confirmed that there were no differences in the global GM and WM measures between the two RR-MS groups (WM: T = −0.85, P = 0.40; GM: T = 0.12, P = 0.94). Finally, VBM analyses showed a significant main effect of group in the bilateral thalamus and right posterior hippocampus (P<0.05, FWE, whole-brain correction and P<0.05, FWE, svc in the hippocampal ROI) (Table S27), although no statistically significant main effect of genotype or any group by genotype interaction was found (P<0.05, FWE, whole-brain correction or P<0.05, FWE, svc in the hippocampal and PCC ROIs) (Table S28–S29).

### fMRI results

#### fMRI regional effects

**Figure 1:**
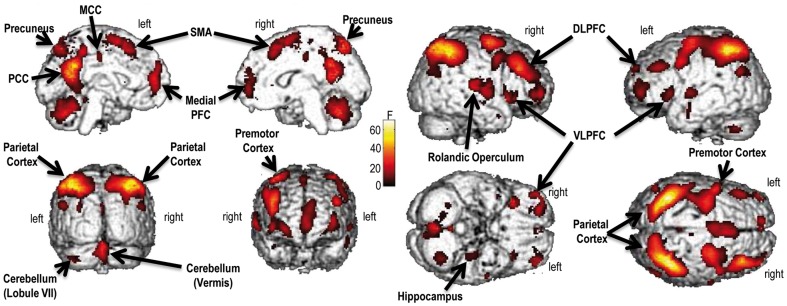
Main effect of task (i.e., encoding, retrieval). The brain regions displayed are those where the activity was modulated by encoding and retrieval visual scenes. Specifically, areas more active during encoding than retrieval were the following: (1) rolandic operculum; (2) posterior cingulate cortex (PCC); (3) precuneus; (4) middle cingulate cortex (MCC); (5) medial prefrontal cortex (PFC); (6) posterior hippocampus. Vice versa, these other regions were more active during retrieval versus encoding: (1) parietal cortex; (2) dorsolateral prefrontal cortex (DLPFC); (3) ventrolateral prefrontal cortex (VLPFC); (4) premotor cortex; (5) cerebellum (vermis and lobule VII); (6) supplementary motor area (SMA). The color bar represents F statistics. Maps are thresholded at P<0.05, Family Wise Error (FWE), whole-brain correction.

**Figure 2:**
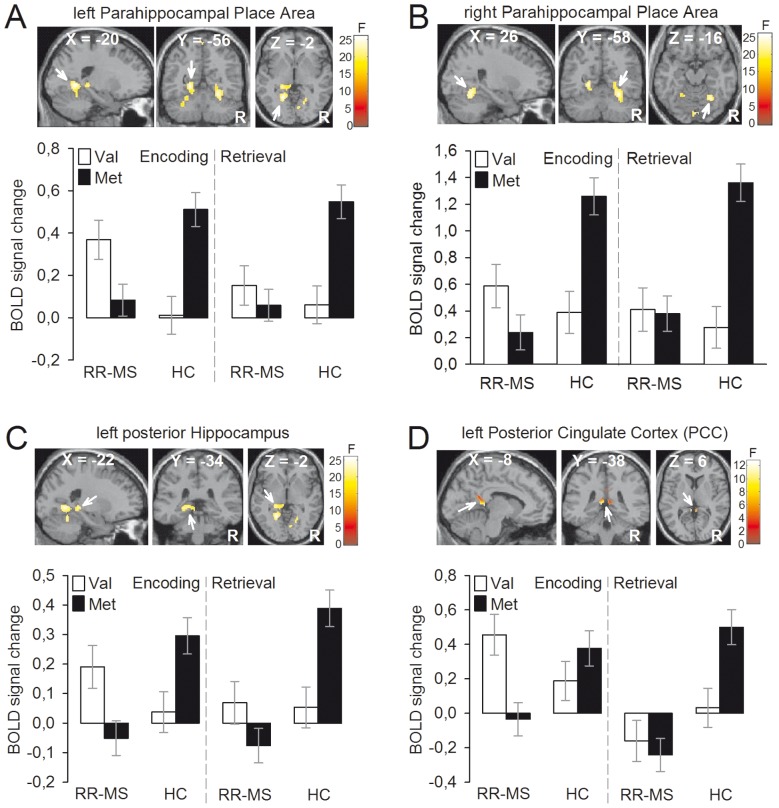
Group (RR-MS, HC) by BDNF genotype (Val^66^, Met^66^) interaction. Left and right parahippocampal place areas (**A–B**), left posterior hippocampus (**C**), and left posterior cingulate cortex (PCC) (**D**) showed greater response in RR-MS Val^66^ homozygous versus RR-MS Met^66^ carriers while the *opposite was true* for HC (i.e., greater response in HC Met^66^ carriers versus HC Val^66^ homozygous). Color bars represent F statistics. Coordinates (X, Y, Z) are in the Montreal Neurological Institute space. Only for a display purpose, maps are thresholded at P<0.001, uncorrected, but results are significant at P<0.05, Family-Wise Error (FWE), whole-brain correction (**A–B**) and small volume correction (svc) (**C–D**). BOLD = blood oxygenated level dependent; R = right hemisphere; RR-MS = Relapsing-Remitting Multiple Sclerosis; HC = Healthy Controls.

Significant effects were found for: (1) the main effect of group (Table S1); (2) the main effect of task (**Fig. 1**, **Table 3**); (3) the group by genotype interaction (**Fig. 2**, Table S3); (4) the group by task interaction (Table S4). In contrast, no statistically significant effect was found for the main effect of genotype (Table S2), the genotype by task interaction (Table S5) and the group by genotype by task interaction (Table S6).

**Table 3:** Brain regions showing a main effect of the Task (Encoding, Retrieval) (see **Figure 1**).

Brain region	Hemisphere	F Local Maxima	MNI coordinates		
			x	y	z
Parietal Cortex	L	70.31*	−36	−52	52
	R	64.93*	38	−56	52
Rolandic Operculum	R	67.30*	40	−16	16
Posterior Cingulate Cortex (PCC) and Precuneus	L	59.62*	−8	−56	16
	R	45.66*	8	−54	16
Middle Cingulate Cortex (MCC)	L	32.25*	−6	−40	50
Dorso lateral Prefrontal Cortex (DLPFC)	L	33.01*	−36	56	2
	R	53.33*	38	32	32
Ventro lateral Prefrontal Cortex (VLPFC)	L	34.68*	−36	16	4
	R	48.58*	32	28	0
Premotor Cortex	L	37.87*	−30	−8	56
	R	42.62*	28	−8	56
Medial Prefrontal Cortex (PFC)	L	42.72*	−4	54	4
Cerebellum (Vermis)	R	38.42*	2	−64	−32
Cerebellum (Lobule VII)	L	28.76*	−34	−64	−46
Supplementary Motor Area (SMA)	L	33.10*	−2	2	62
	R	37.16*	4	18	54
Hippocampus	L	20.30**	−26	−20	−20
	R	17.00**	40	−12	−14

**Legend.** L, left; R, right; MNI, Montreal Neurological Institute;

* P<0.05, Family Wise Error (FWE), whole-brain correction.

** P<0.05, Family Wise Error (FWE), small volume correction (svc).

The ANOVA investigating the main effect of group revealed enhanced PCC activation in HC versus RR-MS patients during both encoding and retrieval (P<0.05, FWE, svc)( Table S1).

Furthermore, a number of regions within a distributed brain network demonstrated a significant main effect of task (P's<0.05, FWE, whole-brain correction; P<0.05, FWE, svc, for ROIs)(**Fig. 1**,**Table 3**).

Of note, the group by genotype analysis revealed an highly significant interaction in the bilateral para-hippocampal place area (PPA), left posterior hippocampus, and PCC (**Fig. 2 A–D** **, top panels**)(Table S3). This interaction was driven by opposite effects of the BDNF genotype in RR-MS patients relative to HC during both encoding and retrieval (i.e., brain responses were greater in RR-MS Val^66^ homozygous versus RR-MS Met^66^ carriers while the reverse was true for HC) (**Fig. 2 A–D** **, bottom panels**).

Finally, a group by task interaction revealed greater PCC activation in RR-MS patients compared to HC for encoding versus retrieval (Table S4).

#### Functional connectivity results

**Figure 3:**
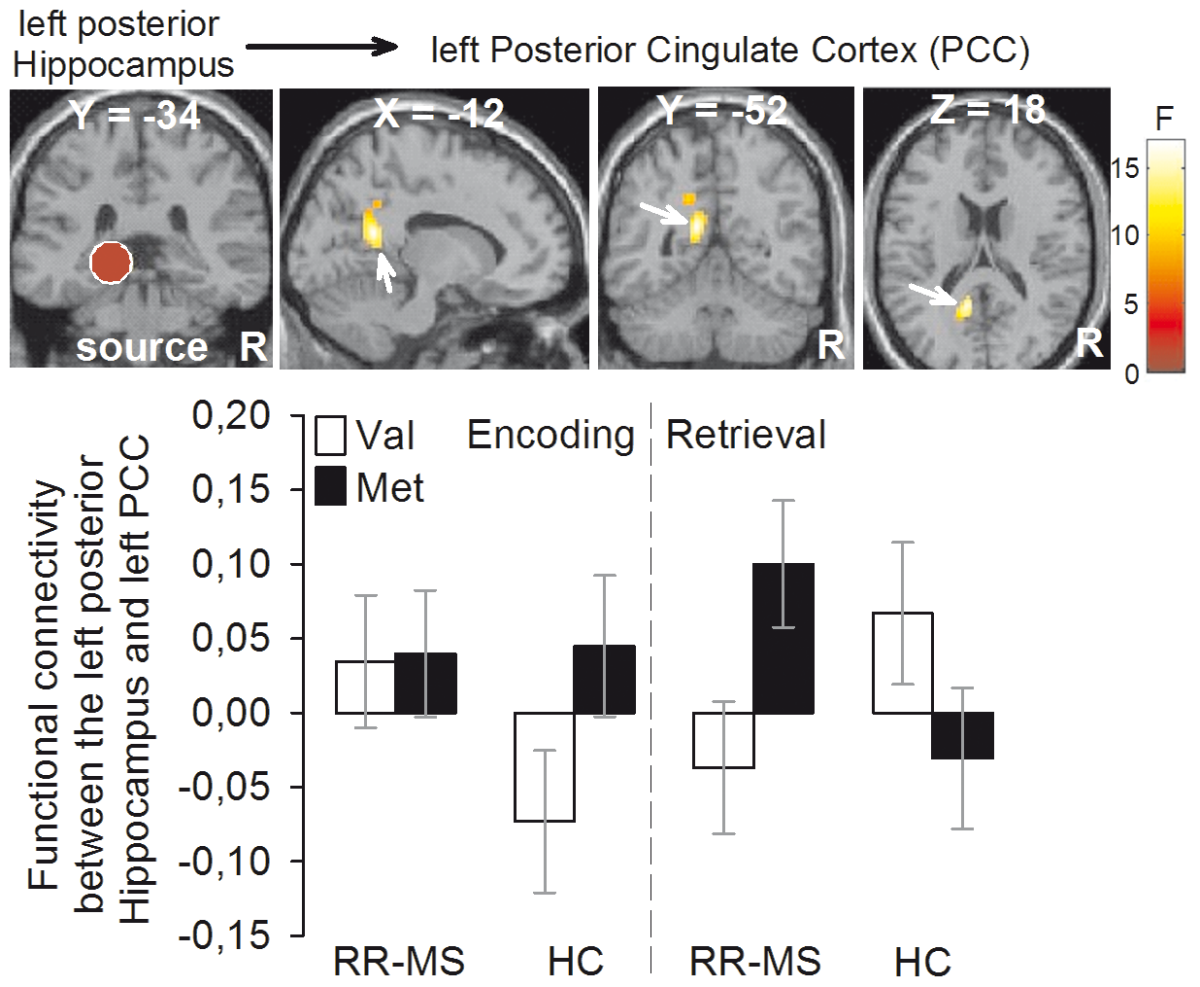
Functional connectivity results (group [RR-MS, HC] by BDNF genotype [Val66, Met66] by task [encoding, retrieval] interaction). The left posterior cingulate cortex displayed greater connectivity with the left hippocampus ‘source’ in RR-MS Met66 carriers vs. RR-MS Val66 homozygous patients during retrieval (but not encoding) while the opposite effect was found in HC (i.e., left hippocampus-PCC connectivity was greater in HC Val66 homozygous vs. HC Met66 carriers during retrieval but not encoding). Color bar represents F statistics. Coordinates (X, Y, Z) are in the Montreal Neurological Institute space. Only for a display purpose, maps are thresholded at P<0.001, uncorrected, but results are significant at P<0.05, Family-Wise Error (FWE), small volume correction (svc). RR-MS = relapsing-remitting multiple sclerosis; HC = healthy controls; BDNF = Brain Derived Neurotrophic Factor; R = right hemisphere.

An enhanced intra-hippocampal connectivity for the main effect of group (i.e., increased connectivity between the left posterior hippocampus ‘source’ and the bilateral anterior hippocampus) was found in RR-MS patients when compared to HC during both encoding and retrieval (Table S7). Increased connectivity between the left posterior hippocampus and the bilateral PCC during retrieval versus encoding was found for the main effect of task (Table S9). In contrast, no statistically significant effect was found for the main effect of genotype (Table S8), the group by genotype interaction (Table S10), the group by task interaction (Table S11) and the genotype by task interaction (Table S12). The ANOVA investigating the group by genotype by task interaction revealed a remarkable effect in the left PCC (**Fig. 3** **, top panels**, Table S13). This results was driven by a greater left hippocampus-PCC connectivity in RR-MS Met^66^ carriers relative to RR-MS Val^66^ homozygous during retrieval (but not encoding) and by an opposite effect in HC (i.e., greater left hippocampus-PCC connectivity in HC Val^66^ homozygous versus HC Met^66^ carriers during retrieval but not encoding)(**Figure 3** **, bottom panels**).

#### fMRI results with behavioral covariates as effect of no interest in the GLM

When including RTs during retrieval, digit symbol scores and visuo-spatial memory measure as covariates of no interest in the GLMs assessing the main effect of group, the main effect of genotype and the group by genotype interaction on activity in single regions and functional connectivity patterns with the left hippocampal seed, we found that functional connectivity analyses were overall unaffected by the inclusion of these covariates (Table S20–S26). In contrast, the regional effect in the PCC for the group by task interaction was statistically more robust in the GLM with the covariates than in the GLM without the covariates (Table S17). The opposite was true for the main effect of group and the group by genotype interaction (i.e., the effect in PCC became statistically not significant after the inclusion of the covariates) (Table S14, S16). The other effects remained unchanged (Table S15, S18, S19).

## Discussion

This study provides new evidence that the BDNF Val^66^Met polymorphism has opposite effects on the hippocampal memory circuit of cognitively unimpaired RR-MS patients and HC. Specifically, we found that RR-MS Val^66^ homozygous, relative to RR-MS Met^66^ carriers, showed greater responses in brain regions of the episodic memory network during both encoding and retrieval and a lack of hippocampal-PCC connectivity during retrieval *but not* encoding (*vice versa* for HC Val^66^ homozygous versus HC Met^66^ carriers). Of note, two different, but complementary, approaches for analyzing fMRI data (i.e., analyses of regional effects and connectivity methods) demonstrated the genotype by diagnosis interaction.

We meticulously selected, from a sample of 50 patients, only those RR-MS patients (n = 26) with a mild disease (i.e., EDDS range: 1–2.5) and who were also off-medication and cognitively preserved, as evaluated by a detailed neuropsychological assessment. These strict inclusion criteria were adopted to make the fMRI results fully interpretable (e.g., a cognitive deficit may have prevented the correct execution of the task) and to eliminate biases associated with advanced disease stages and pharmacological treatments. The importance of studying the effect of a genotype even in small, although well-selected, samples is that the results obtained could represent useful biomarkers of memory function in RR-MS. Nonetheless, we acknowledge that this is a pilot study that requires replication in bigger samples and other groups of MS patients (e.g., primary and secondary progressive MS).

### 

#### Effect of the BDNF genotype on brain regional responses

The increased BOLD activations within single regions of the episodic memory system (i.e., hippocampus, PPA, and PCC) of RR-MS Val^66^ homozygous versus RR-MS Met^66^ carriers may reflect gene-specific compensatory mechanisms aimed at maintaining normal behavioral performances during the fMRI task. This interpretation is strongly supported by previous studies investigating compensatory adaptations in sensory-motor, cognitive and emotional brain regions of MS patients [6]–[12], [56]–[59].

Recently, a study demonstrated enhanced activations in the hippocampal system of MS patients without neuropsychological deficits and reduced hippocampal responses in cognitively impaired MS patients during an episodic memory task [13]. This study concluded that hippocampal hyperactivations may represent a brain functional adaptation that would be overridden when cognitive deficits become manifest at the clinical level [13]. Similarly, we found that including fMRI behavioral measures and neuropsychological scores assessing information processing speed and episodic memory, significantly affected the regional activations in the PCC.

The critical role played by the BDNF genotype in modulating the hippocampal memory function has also been demonstrated in HC. In particular, HC Met^66^ carriers, relative to HC Val^66^ homozygous, maintained normal behavioral performances via an enhanced hippocampal/parahippocampal response during two tasks assessing different types of episodic memory (i.e., item- and relational-episodic memory) [32]. Furthermore, one of the first study investigating the effect of the BDNF Val^66^Met polymorphism at a brain and behavioral level showed that lower accuracy on memory performances were associated with reduced hippocampal activity in HC Met^66^ carriers, relative to HC Val^66^ homozygous [22].

The brain areas within the hippocampal system that showed the significant genotype by group interaction are known to be fundamental regions for episodic memory. Specifically, the parahippocampal place area (PPA) is engaged in spatial navigation and encoding the “geometry” of complex visual scenes [40]–[41]. Furthermore, the hippocampus *itself* is the key region where the acquisition of memories takes place via cellular mechanisms dependent on the BDNF release [3], [24]. Our findings of a posterior localization and laterality (i.e., left side) of the hippocampal activation may depend on the nature of the stimuli used (i.e., visual scenes), as also showed by a previous study that used a similar paradigm [22].

Last but not least, there is converging evidence from animal and human research that the posterior cingulate cortex (PCC) is a critical area for the retrieval of episodic memory information as well as for spatial navigation and scene “reconstruction” [43], [72].

#### Effect of the BDNF genotype on hippocampal functional connectivity

In addition to the genotype effect on responses of single brain regions, we found a remarkable group by genotype by task interaction on functional connectivity data. In particular, RR-MS Val^66^ homozygous and HC Met^66^ carriers displayed a lack of connectivity between the left posterior hippocampus and PCC during retrieval *but not* encoding. These results suggest that an abnormal pattern of functional connectivity within the hippocampal memory system may be at the basis of the compensatory hyperactivations in single brain regions showed by RR-MS Val^66^ homozygous and HC Met^66^ carriers and discussed in **section 5.1**.

However, it should be highlighted that we found a group by genotype interaction for the brain regional responses while a group by genotype by task interaction was detected for connectivity analyses. This means that our interpretation of the link between abnormal functional connectivity patterns and compensatory hyperactivations in single regions can be applied to the retrieval- *but not* encoding-phase of the fMRI task. It is likely that the abnormal connectivity patterns in RR-MS Val66 homozygous and HC Met66 carriers became evident only when episodic memory networks are working at their maximal level, i.e., during retrieval of visual stimuli rather than passive encoding. Alternatively, it may be that our data were under-powered to detect subtle connectivity effects during the encoding phase of the task. Whichever the explanation, it is of note that a clear connectivity pattern emerged between the PCC and hippocampus, two key regions that have been strongly implicated in various forms of anterograde and retrograde amnesia [72].

The functional importance of PCC-hippocampal neural pathways is also highlighted by disconnection studies in rats showing that PCC and hippocampus require each other to support spatial learning [72]. In humans, the reduction of PCC metabolism is the earliest brain functional abnormality displayed by patients with Alzheimer's disease and is thought to depend on the deafferentation of PCC from hippocampal inputs [72].

Hence, it is likely that the demyelination and/or axonal loss typically associated with RR-MS alters the ‘communications’ between the hippocampus and PCC and, in turn, triggers compensatory responses (i.e., over-activations) within these regions. As MS progresses, the increase of the white-matter damage over a certain threshold would abolish such responses, in accordance with a recent study combining fMRI and diffusion tensor imaging (DTI) data [14].

#### Mechanisms of the BDNF genotype by RR-MS interaction

Another critical point regards the nature of the biological mechanisms underlying the genotype by group interaction. Although addressing this issue is beyond the scope of the present study, a possibility is that the autoimmune changes associated with MS (e.g., T-cells proliferation, secretion of interleukins and neurotrophic factors including the BDNF) interact, at a molecular and cellular level, with the pre-existing BDNF genotype. In particular, it may be that the detrimental effect of the Met^66^ allele, described in animal models and *in vitro* (e.g., altered BDNF intracellular trafficking), is reversed and thus becomes beneficial in RR-MS patients. In line with this hypothesis, we found that the BDNF mRNA levels in the peripheral blood cells were higher in RR-MS Met^66^ carriers relative to RR-MS Val^66^ homozygous and HC (both HC Met^66^ carriers and HC Val^66^ homozygous) [45]. In a larger group of MS patients, the Met^66^ allele has also been associated with preserved grey-matter volume, reduced total lesion load, and higher scores in a test assessing information processing speed (but see **section 5.4.** for further discussion on this issue) [31]. These results were similar to previous findings showing a protective effect of the Met^66^ allele against the cognitive dysfunctions that appear in Systemic Lupus Erythematosus, a severe autoimmune disorder as MS [73]. Certainly, the mechanisms underlying the effect of the BDNF Val^66^Met polymorphism on the episodic memory system of RR-MS patients require additional investigations at a molecular and cellular level.

Nonetheless, it is interesting that a reversed effect of the Val^66^Met polymorphism has also been described in other disorders in which BDNF plays an important pathophysiological role. For example, an fMRI study in anxious and depressed adolescents demonstrated a significant group by genotype interaction on the amygdala response to affective stimuli [74]. Furthermore, there is evidence that the way in which the BDNF Met^66^Val polymorphism modulates episodic memory and some structural brain measures (i.e., cortical thickness and fractional anisotropy [FA]) depends on subjects age [75]. Specifically, the Met^66^ allele has been associated, in young people, with impaired episodic memory and reduced cortical thickness and FA, a measure of white matter integrity [75]. In contrast, elderly people carrying the Met^66^ allele display the opposite effects (i.e., better memory performances and increased cortical thickness and FA values)(*vice versa* for the young and old people homozygous for the Val^66^ allele) [75].

#### Relation between fMRI and structural findings

The issue of how current results relate to current and previous MRI structural findings should be now discussed, although it is always difficult to interpret fMRI results at the light of structural MRI studies, given that little is known about the links between brain function and the underlying neuroanatomy.

In our previous research, we found a genotype by group interaction on the global grey-matter (GM) volume that was driven by *reduced* GM in RR-MS Met^66^ carriers relative to RR-MS Val^66^ homozygous and HC (both HC Met^66^ carriers and HC Val^66^ homozygous) [27]. However, as anticipated in **section 5.3.**, other authors found *increased* and *not reduced* GM in MS Met^66^ carriers relative to MS Val^66^ homozygous, although their sample did not include HC Met^66^ carriers and HC Val^66^ homozygous that would have allowed to studying the group by genotype interaction [31]. Likewise, a recent VBM study in MS patients reported increased GM within the parahippocampal gyrus, precuneus, and other regions in MS Met^66^ carriers versus MS Val^66^ homozygous, although results did not survive statistical correction for the whole-brain (i.e., the significance level was P<0.001, uncorrected) [30]. Overall, we believe that part of these inconsistencies may be explained by different inclusion criteria (e.g., enrollment of patients with secondary progressive MS and/or patients under disease modifying therapies), different sample sizes and different methodological approaches to calculate the grey-matter volume.

Nonetheless, here, we found that the interactive effect of BDNF genotype and MS was evident for the *function* but not the *structure* of the hippocampus. This may depend on the intrinsic ‘on-line’ nature of fMRI that is better suited than structural methods to detect fast-acting effects of the BDNF, a neurotrophin known to elicit rapid neuronal signaling in addition to classic slow modulatory effects [21], [23].

## Conclusions

In summary, we found that a common BDNF polymorphism (Val^66^→Met^66^ substitution) was associated with opposite effects on the hippocampal memory system of RR-MS patients and HC. Specifically, relative to HC Val^66^ homozygous, HC Met^66^ carriers displayed a lack of hippocampal connectivity change and enhanced ‘compensatory’ activations in single regions during the retrieval phase of the episodic memory task. Of note, reversed effects were found in RR-MS patients. Given previous evidence showing increased mRNA production in RR-MS Met^66^ carriers [45], we hypothesize that MS-related mechanisms (e.g., autoimmunity) may be responsible of reverting the detrimental effect of the Met^66^ allele.

Enhancing our understanding of how genetic factors contribute to memory function in MS may in future guarantee a better management of cognitive deficits, with clear benefits for patients.

## Supporting Information

File S1 **Combined file containing all supporting information tables.** Table S1: L, left; R, right; MNI, Montreal Neurological Institute; ^a^P<0.05, Family Wise Error (FWE), small volume correction (svc). Table S2 (no legend). Table S3: L, left; R, right; MNI, Montreal Neurological Institute; ^a^P<0.05, Family Wise Error (FWE), small volume correction (svc); ^b^P<0.05, Family Wise Error (FWE), whole-brain correction. Table S4: L, left; R, right; MNI, Montreal Neurological Institute; ^a^P<0.05, Family Wise Error (FWE), small volume correction (svc). Table S5 (no legend). Table S6 (no legend). Table S7: L, left; R, right; MNI, Montreal Neurological Institute; ^a^P<0.05, Family Wise Error (FWE), small volume correction (svc). Table S8 (no legend). Table S9 (no legend). Table S10: L, left; R, right; MNI, Montreal Neurological Institute; ^a^P<0.05, Family Wise Error (FWE), small volume correction (svc). Table S11 (no legend). Table S12 (no legend). Table S13: L, left; MNI, Montreal Neurological Institute. ^a^P<0.05, Family Wise Error (FWE), small volume correction (svc). Table S14 (no legend). Table S15 (no legend). Table S16: L, left; R, right; MNI, Montreal Neurological Institute; ^a^P<0.05, Family Wise Error (FWE), small volume correction (svc); ^b^P<0.05, Family Wise Error (FWE), whole-brain correction. Table S17: L, left; R, right; MNI, Montreal Neurological Institute; ^a^P<0.05, Family Wise Error (FWE), whole-brain correction. ^b^P<0.05, Family Wise Error (FWE), small volume correction (svc). Table S18 (no legend). Table S19 (no legend). Table S20: L, left; R, right; MNI, Montreal Neurological Institute; ^a^P<0.05, Family Wise Error (FWE), small volume correction (svc). Table S21 (no legend). Table S22: L, left; R, right; MNI, Montreal Neurological Institute; ^a^P<0.05, Family Wise Error (FWE), small volume correction (svc). Table S23 (no legend). Table S24 (no legend). Table S25 (no legend). Table S26: L, left; MNI, Montreal Neurological Institute. ^a^P<0.05, Family Wise Error (FWE), small volume correction (svc). Table S27: L, left; MNI, Montreal Neurological Institute. ^a^P<0.05, Family Wise Error (FWE), whole-brain correction. ^b^P<0.05, Family Wise Error (FWE), small volume correction (svc). Table S28 (no legend). Table S29 (no legend).
(ZIP)
