# Supplementary material for: The BDNF Val66Met Polymorphism Has Opposite Effects on Memory Circuits of Multiple Sclerosis Patients and Controls
Source: PLoS One. 2013 Apr 11;8(4):e61063. doi: 10.1371/journal.pone.0061063 (PMC3623818; doi:10.1371/journal.pone.0061063)
Supplement: File S1 — Combined file containing all supporting information tables. Table S1: L, left; R, right; MNI, Montreal Neurological Institute; aP<0.05, Family Wise Error (FWE), small volume correction (svc). Table S2 (no legend). Table S3: L, left; R, right; MNI, Montreal Neurological Institute; aP<0.05, Family Wise Error (FWE), small volume correction (svc); bP<0.05, Family Wise Error (FWE), whole-brain correction. Table S4: L, left; R, right; MNI, Montreal Neurological Institute; aP<0.05, Family Wise Error (FWE), small volume correction (svc). Table S5 (no legend). Table S6 (no legend). Table S7: L, left; R, right; MNI, Montreal Neurological Institute; aP<0.05, Family Wise Error (FWE), small volume correction (svc). Table S8 (no legend). Table S9 (no legend). Table S10: L, left; R, right; MNI, Montreal Neurological Institute; aP<0.05, Family Wise Error (FWE), small volume correction (svc). Table S11 (no legend). Table S12 (no legend). Table S13: L, left; MNI, Montreal Neurological Institute. aP<0.05, Family Wise Error (FWE), small volume correction (svc). Table S14 (no legend). Table S15 (no legend). Table S16: L, left; R, right; MNI, Montreal Neurological Institute; aP<0.05, Family Wise Error (FWE), small volume correction (svc); bP<0.05, Family Wise Error (FWE), whole-brain correction. Table S17: L, left; R, right; MNI, Montreal Neurological Institute; aP<0.05, Family Wise Error (FWE), whole-brain correction. bP<0.05, Family Wise Error (FWE), small volume correction (svc). Table S18 (no legend). Table S19 (no legend). Table S20: L, left; R, right; MNI, Montreal Neurological Institute; aP<0.05, Family Wise Error (FWE), small volume correction (svc). Table S21 (no legend). Table S22: L, left; R, right; MNI, Montreal Neurological Institute; aP<0.05, Family Wise Error (FWE), small volume correction (svc). Table S23 (no legend). Table S24 (no legend). Table S25 (no legend). Table S26: L, left; MNI, Montreal Neurological Institute. aP<0.05, Family Wise Error (FWE), small volume corre [file pone.0061063.s001.zip › Table S5.doc]

**Supplementary Table 5.** Brain regions showing a BDNF Genotype (Val^66^, Met^66^) X Task (Encoding, Retrieval) Interaction.

| **Brain region** | **Hemisphere** | **F Local Maxima** | **MNI coordinates** | | |
| --- | --- | --- | --- | --- | --- |
| None | None | None | None | None | None |
